# *Thanks to our peer reviewers*

**DOI:** 10.1002/lrh2.10042

**Published:** 2017-09-15

The publication of Issue 4 marks the completion of **Volume 1** of *Learning Health Systems*. Reaching this milestone is thrilling for all of us who have been involved in getting the journal launched. We are keenly aware that this achievement would not have happened without the dedicated efforts and insightful comments of those individuals who accepted invitations to review submitted articles. In spite of busy schedules and full commitments, these individuals found the time and energy to contribute their expertise and suggestions to our authors to help ensure that their papers met (and often exceeded) this journal's high standards for publication. *Please accept our sincere gratitude for a job well done!*

Charles P. Friedman, *Editor in Chief*

## Reviewers for Volume 1 LHS Journal

John Ainsworth (United Kingdom)
Christel Anderson (United States)
Holt Anderson (United States)
Ruth Anderson (United States)
Theodoros Arvanitis (United Kingdom)
Bo Borgnakke (United States)
Hendriek Boshuizen (Netherlands)
Iain Buchan (United Kingdom)
Michael Cantor (United States)
Christopher Chute (United States)
Laura Crawford (United States)
Vasa Curcin (United Kingdom)
Jennifer Dixon (United Kingdom)
Stephen Duffield (Australia)
Patricia Dykes (United States)
Lynn Etheredge (United States)
Thomas Foley (United Kingdom)
Oliver Gadabu (United States)
Mari Greenberger (United States)
Terry Hannan (United Kingdom)
Wolfgang Kuchinke (Germany)
Rebecca Kush (United States)
Harold Lehmann (United States)
Christopher Longhurst (United States)
David McCallie (United States)
Mark McGilchrist (United Kingdom)
Meryt McGindley (United States)
Kenneth Mandl (United States)
Erik Mayer (United Kingdom)
Amalia Miller (United States)
Megan Moncur (United States)
Stephanie Morain (United States)
Sally Okun (United States)
Leon Osterweil (United States)
Adam Porter (United States)
William Rouse (United States)
Sonya Satveit (Canada)
Philip Scott (United Kingdom)
Ben Shneiderman (United States)
Jonathan Silverstein (United States)
Sunnie Southern (United States)
Kevin Sullivan (United States)
Joshua Vest (United States)
Jim Walker (United States)

